# 3,4,5-Trichloroaniline Nephrotoxicity *in Vitro*: Potential Role of Free Radicals and Renal Biotransformation

**DOI:** 10.3390/ijms151120900

**Published:** 2014-11-13

**Authors:** Christopher Racine, Dakota Ward, Dianne K. Anestis, Travis Ferguson, Deborah Preston, Gary O. Rankin

**Affiliations:** 1 Department of Pharmacology, Physiology and Toxicology, Joan C. Edwards School of Medicine, Marshall University, Huntington, WV 25755, USA; E-Mails: racine@live.marshall.edu (C.R.); ward210@live.marshall.edu (D.W.); anestis@marshall.edu (D.K.A.); ferguson125@live.marshall.edu (T.F.); 2 Department of Pediatrics, Joan C. Edwards School of Medicine, Marshall University, Huntington, WV 25755, USA; E-Mail: preston28@marshall.edu

**Keywords:** 3,4,5-Trichloroaniline, nephrotoxicity, kidney, Fischer 344 rat, *in vitro*, antioxidants

## Abstract

Chloroanilines are widely used in the manufacture of drugs, pesticides and industrial intermediates. Among the trichloroanilines, 3,4,5-trichloroaniline (TCA) is the most potent nephrotoxicant *in vivo*. The purpose of this study was to examine the nephrotoxic potential of TCA *in vitro* and to determine if renal biotransformation and/or free radicals contributed to TCA cytotoxicity using isolated renal cortical cells (IRCC) from male Fischer 344 rats as the animal model. IRCC (~4 million cells/mL; 3 mL) were incubated with TCA (0, 0.1, 0.25, 0.5 or 1.0 mM) for 60–120 min. In some experiments, IRCC were pretreated with an antioxidant or a cytochrome P450 (CYP), flavin monooxygenase (FMO), cyclooxygenase or peroxidase inhibitor prior to incubation with dimethyl sulfoxide (control) or TCA (0.5 mM) for 120 min. At 60 min, TCA did not induce cytotoxicity, but induced cytotoxicity as early as 90 min with 0.5 mM or higher TCA and at 120 min with 0.1 mM or higher TCA, as evidenced by increased lactate dehydrogenase (LDH) release. Pretreatment with the CYP inhibitor piperonyl butoxide, the cyclooxygenase inhibitor indomethacin or the peroxidase inhibitor mercaptosuccinate attenuated TCA cytotoxicity, while pretreatment with FMO inhibitors or the CYP inhibitor metyrapone had no effect on TCA nephrotoxicity. Pretreatment with an antioxidant (α-tocopherol, glutathione, ascorbate or *N*-acetyl-l-cysteine) also reduced or completely blocked TCA cytotoxicity. These results indicate that TCA is directly nephrotoxic to IRCC in a time and concentration dependent manner. Bioactivation of TCA to toxic metabolites by CYP, cyclooxygenase and/or peroxidase contributes to the mechanism of TCA nephrotoxicity. Lastly, free radicals play a role in TCA cytotoxicity, although the exact nature of the origin of these radicals remains to be determined.

## 1. Introduction

Chloroanilines are commonly used as chemical intermediates to manufacture dyes, agricultural chemicals, drugs and industrial compounds. Exposure to chloroanilines can occur in occupational settings, through the release or formation during the metabolism of compounds in mammals [1,2,3] or by the degradation of pesticides in the environment [1,4,5,6]. In addition, the detection of chloroanilines in human urine or blood can be used as a biomarker for exposure to chloroaniline-based pesticides [7,8,9,10]. The toxicity associated with exposure to mono- and dichloroanilines includes hematotoxicity (e.g. methemoglobinemia or anemia) [11,12,13], splenotoxicity [11,14], hepatotoxicity [15,16,17] and nephrotoxicity [16,18,19]. Because of their adverse health effects and release into the environment in agricultural areas following the breakdown of pesticides, chloroanilines are considered priority pollutants in environmental risk assessments [20,21].

Trichloroanilines have similar uses as the mono- and dichloroanilines, including use in drug development [22,23,24], dye manufacturing [25] and production of agricultural agents [26]. While the potential adverse health effects of mono- and dichloroanilines have been studied in some detail, little information is available about the toxicity induced by trichloroanilines, including the nephrotoxic potential of trichloroanilines or their mechanisms of inducing nephrotoxicity. Lo *et al.* examined the *in vivo* and *in vitro* effects of four trichloroanilines (2,3,4-, 2,4,5-, 2,4,6- and 3,4,5-trichloroaniline) on the renal function of male Fischer 344 rats [27]. They noted that of the four trichloroanilines tested, 3,4,5-trichloroaniline (TCA) had the greatest nephrotoxic potential *in vivo* as evidenced by oliguria, increased kidney weight, elevated blood urea nitrogen concentration and altered renal organic ion accumulation. *In vitro*, TCA was also the most potent nephrotoxicant of the four trichloroanilines tested, decreasing tetraethylammonium accumulation by renal cortical slices at 1.0 µM concentration [27].

Although it is known that metabolites of mono- and dichloroanilines are toxic to the kidney *in vivo* and *in vitro* [28,29,30,31], no studies have examined the role of biotransformation in trichloroaniline nephrotoxicity. It is also unknown if the kidney bioactivates parent chloroanilines to nephrotoxic metabolites, or if the parent chloroaniline is toxic to the kidney without bioactivation. The purpose of this study was to begin to examine the role of biotransformation of a trichloroaniline in the nephrotoxicity it produces *in vitro* as well as determine if free radicals contributed to the cytotoxicity. TCA was selected for study because it is the most potent trichloroaniline nephrotoxicant *in vivo* and *in vitro*. The Fischer 344 rat was selected as the animal model because our previous studies with chloroaniline-induced nephrotoxicity have been conducted in this animal model. The inhibitor pretreatments, concentrations and times selected for study were based on previous reports [32,33,34,35,36,37,38,39].

## 2. Results

### 2.1. Time and Concentration Cytotoxicity Studies

**Figure 1:**
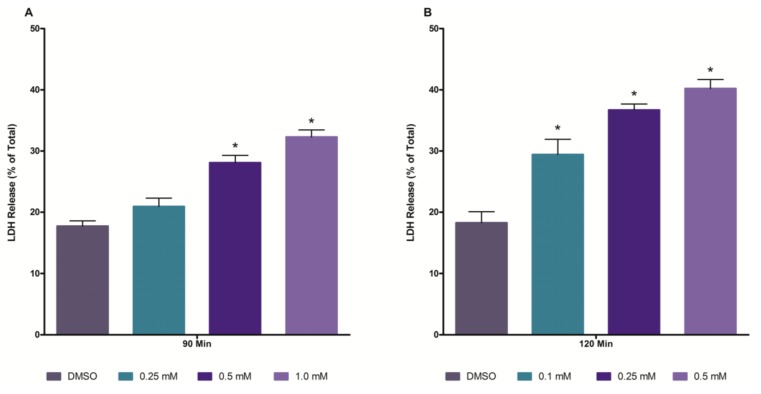
Cytotoxic effects of TCA at 90 min (**Panel A**) and 120 min (**Panel B**) in isolated renal cortical cells (IRCC). An asterisk indicates significantly different from the dimethyl sulfoxide (DMSO) control group value, *p* < 0.05.

To obtain information concerning the nephrotoxic potential of TCA in isolated renal cortical cells (IRCC), a concentration response study was performed at 60, 90 and 120 min. IRCC exposed to TCA at concentrations up to 1.0 mM for 60 min did not exhibit any cytotoxicity (data not shown). A 90 min exposure to TCA at a concentration of 0.5 mM or higher induced cytotoxicity (increased LDH release), while at 120 min of exposure, cytotoxicity was evident at a concentration of 0.1 mM TCA or higher (Figure 1). Based on these findings, a concentration of 0.5 mM and an exposure time of 120 min were selected for use in the antioxidant and inhibitor pretreatment studies.

### 2.2. Effects of Antioxidants on 3,4,5-Trichloroaniline (TCA) Cytotoxicity

**Figure 2:**
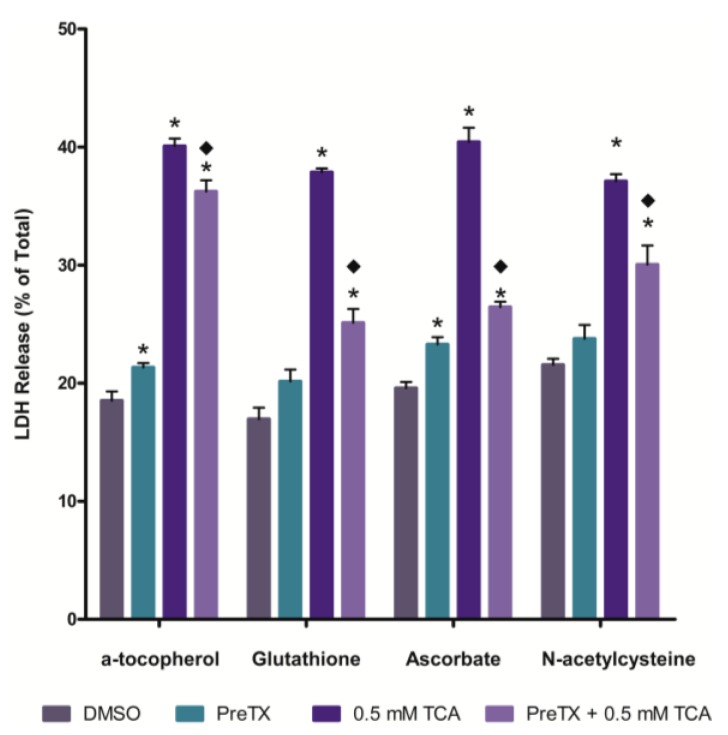
Effect of antioxidant pretreatment on TCA cytotoxicity at 120 min. An asterisk indicates significantly different from the DMSO control group value, *p* < 0.05. A diamond indicates significantly different from the 0.5 mM TCA alone value, *p* < 0.05.

The effects of pretreating IRCC with an antioxidant on TCA cytotoxicity was examined next (Figure 2). All four antioxidants (α-tocopherol, ascorbate, glutathione and *N*-acetyl-l-cysteine) provided at least some degree of attenuation of TCA cytotoxicity. Glutathione and ascorbate were most effective and α-tocopherol was the least effective at attenuating TCA cytotoxicity.

### 2.3. Effects of Cytochrome P450 (CYP) and Flavin-containing Monooxygenase (FMO) Inhibition

**Figure 3:**
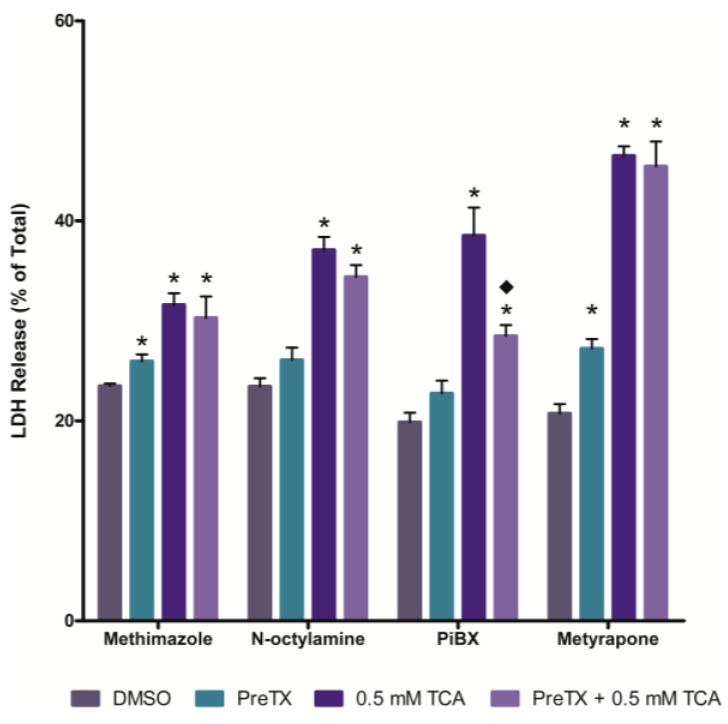
Effect of flavin-containing monooxygenase (FMO) or cytochrome P450 (CYP) inhibition on TCA cytotoxicity at 120 min. An asterisk indicates significantly different from the DMSO control group value, *p* < 0.05. A diamond indicates significantly different from the 0.5 mM TCA alone value, *p* < 0.05.

The effects of inhibiting cytochrome P450 (CYP) and FMO activity on TCA cytotoxicity was examined using nonselective CYP (piperonyl butoxide (PiBX) and metyrapone) and FMO (methimazole and *N*-octylamine) inhibitors. Inhibition of CYPs with PiBX, but not metyrapone, attenuated TCA cytotoxicity (Figure 3). Inhibition of FMOs with either methimazole or *N*-octylamine had no effect on TCA induced cell death (Figure 3).

### 2.4. Effects of Cyclooxygenase and Peroxidase Inhibition

**Figure 4:**
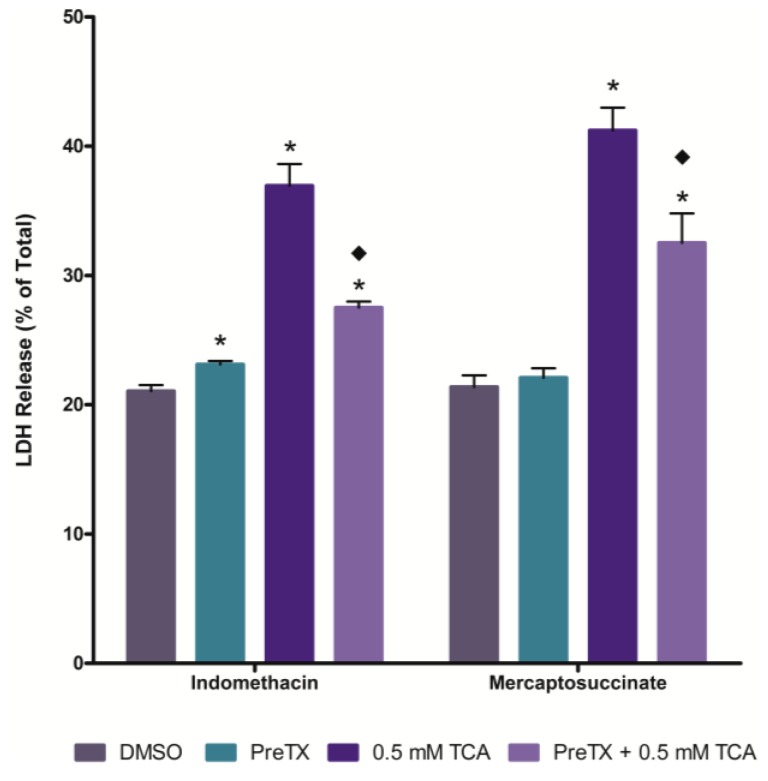
Effect of cyclooxygenase or peroxidase inhibition on TCA cytotoxicity at 120 min. An asterisk indicates significantly different from the DMSO control group value, *p* < 0.05. A diamond indicates significantly different from the 0.5 mM TCA alone value, *p* < 0.05.

The effect of inhibiting the cyclooxygenase activity of prostaglandin H synthase on TCA cytotoxicity was determined using indomethacin pretreatment, while mercaptosuccinate was used as a general peroxidase inhibitor. Both indomethacin pretreatment and mercaptosuccinate pretreatment reduced TCA cytotoxicity (Figure 4).

## 3. Discussion

This study is the first report to demonstrate the direct cytotoxic effects of TCA on the kidney. In a previous study, the *in vitro* effects of TCA on organic ion transport by renal cortical slices from male Fischer 344 rats suggested that TCA could affect renal function, decreasing organic cation accumulation at concentrations as low as 1.0 µM, and affecting both organic anion and cation accumulation at 1.0 mM [27]. However, TCA is a weakly basic compound. Thus, the possibility existed that the effects seen at µM concentrations of TCA in the work by Lo *et al.* [27], were more related to interactions at the organic cation transporter level than cytotoxicity, and that cytotoxicity was not observed until TCA concentrations reached mM levels. Results from the present study clearly demonstrate that TCA can induce cytotoxicity at µM concentrations, as evidenced by increased LDH release at concentrations of TCA as low as 100 µM at 120 min, and that TCA induces cytotoxicity in a time and concentration dependent manner.

**Figure 5:**
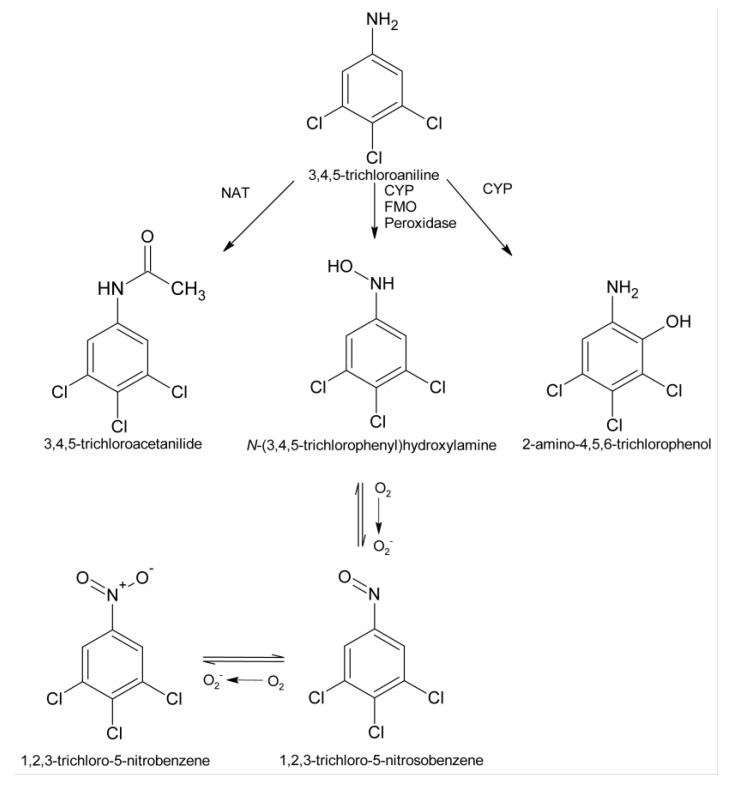
Potential metabolic pathways for TCA. NAT, *N*-acetyltransferase; CYP, cytochrome P450; FMO, flavin-containing monooxygenase.

The ability of the various inhibitors used in this study to attenuate TCA cytotoxicity suggests that metabolites of TCA contribute to TCA nephrotoxicity *in vitro*. The biotransformation of TCA has only been reported in fish [40]. However, based on studies of the metabolism of other chloroanilines in rats [41,42,43,44], a potential biotransformation pathway for TCA can be proposed which includes acetylation, *N*-oxidation and aromatic ring oxidation (Figure 5).

*N*-Acetylation is catalyzed by cytosolic *N*-acetyltransferase enzymes, and it is unlikely that any of the pretreatments used in this study would alter this biotransformation reaction. In addition, acetylation of chloroanilines produces chloroacetanilides, which have greatly reduced nephrotoxic potential *in vivo* [45]. Thus, it is unlikely that acetylation would be a mechanism for bioactivation of 3,4,5-trichloroaniline.

Oxidation of the aromatic ring to produce 2-amino-4,5,6-trichlorophenol would be a potential bioactivation mechanism for TCA, as many aminophenols are known nephrotoxicants [28,29,32,46,47]. Production of an aminophenol metabolite of TCA would be catalyzed by CYPs, as McMillan *et al.* found that aromatic ring oxidation of 3,4-dichloroaniline was catalyzed by CYPs [43,44]. Whether 2-amino-4,5,6-trichlorophenol is produced in the kidney from TCA and contributes to TCA nephrotoxicity remains to be determined. However, a structurally-related metabolite, 2-amino-4,5-dichlorophenol, is directly toxic renal cortical slices from male Fischer 344 rats [48]. Attenuation of TCA cytotoxicity by the CYP inhibitor PiBX suggests that aromatic ring oxidation may be a potential route of TCA bioactivation. Nonetheless, the inability of another general CYP inhibitor, metyrapone, to reduce TCA cytotoxicity indicates that further study is needed to clarify which CYPs may contribute to TCA bioactivation to toxic metabolites and the role of 2-amino-4,5,6-trichlorophenol in TCA nephrotoxicity. The use of additional, more specific CYP inhibitors will help define the role and nature of the CYPs contributing to the production of toxic metabolites in TCA metabolism.

*N*-Oxidation can be catalyzed by several enzyme systems, including CYPs, FMOs, prostaglandin H synthase and peroxidases [44,49,50,51,52,53,54]. The *N*-oxidation pathway has the potential to lead to metabolites that could damage cells via multiple pathways (Figure 5). Metabolites, such as the *N*-hydroxyl and nitroso metabolites can redox cycle to produce reactive oxygen species and other free radicals, which can damage membranes and lead to oxidative stress [13,55]. In addition, *N*-hydroxyl and nitroso metabolites have the ability to be further activated to form covalent bonds with cellular nucleophiles to alter cellular function and led to cell death [56,57,58]. The ability of PiBX, indomethacin and mercaptosuccinate, but not methimazole or *N*-octylamine, to attenuate TCA cytotoxicity suggests that multiple enzyme systems may be involved in bioactivating TCA to toxic metabolites via the *N*-oxidation pathway, but renal FMOs do not appear to contribute to this bioactivation mechanism. That *N*-oxidation would contribute to TCA cytotoxicity is not completely surprising, as 3,4-dichlorophenylhydroxylamine, the *N*-oxidation metabolite of 3,4-dichloroaniline, is a known nephrotoxicant *in vitro* to rat renal cortical slices [31].

The ability of the four antioxidants to attenuate TCA cytotoxicity suggests that free radicals contribute to the mechanism of renal injury induced by TCA. These free radicals may be produced during the oxidation of an aromatic amine or its metabolites [59], or as a consequence of redox cycling of aminophenol or phenylhydroxylamine/nitrosobenzene metabolites to produce reactive oxygen species and oxidative stress [55,60]. Further work is required to determine if either or both of these potential pathways explains the mechanism of protection by antioxidants on TCA cytotoxicity. Preliminary work with another chloroaniline, 3,5-dichloroaniline, suggests that oxidative stress may not contribute to the *in vitro* nephrotoxicity induced by 3,5-dichloroaniline (unpublished data), but studies with TCA have not been conducted to determine how free radicals contribute to TCA nephrotoxicity *in vitro* and are necessary to define the exact role of free radicals in TCA nephrotoxicity.

It is interesting to note that α-tocopherol, a lipophilic compound, was the weakest of the four antioxidants in attenuating TCA nephrotoxicity. It would be expected that α-tocopherol would enter IRCC via a passive diffusion mechanism, while renal transporters can promote the accumulation of ascorbate, glutathione and *N*-acetyl-l-cysteine [61,62,63]. Glutathione can also be accumulated in proximal tubular cells via processing at the luminal membrane and re-synthesis from the accumulated amino acids. Thus, higher intracellular concentrations of ascorbate, glutathione and *N*-acetyl-l-cysteine may be achieved in IRCC as compared to α-tocopherol. In addition, while all of the antioxidants have the ability to scavenge and detoxify a variety of free radicals and reactive oxygen and nitrogen species to varying degrees [64], α-tocopherol associates with membranes and appears to have a primary role in preventing lipid peroxidation, which can lead to cell death. Thus, if TCA does not induce oxidative stress and lipid peroxidation as its primary mechanism for inducing nephrotoxicity, α-tocopherol would be expected to be less effective than the other antioxidants used in this study. However, additional studies are needed to clarify the role of oxidative stress and lipid peroxidation in TCA nephrotoxicity.

## 4. Experimental Section

### 4.1. Experimental Animals

Male Fischer 344 rats (220–280 g) from Hilltop Lab Animals (Scottdale, PA, USA) were used for all experiments. All animals were kept under controlled environments consisting of regulated light cycle (on 12 h, off 12 h), temperature (21–23 °C), and humidity (40%–55%) and were housed in standard plastic cages (two rats per cage). Animals were allowed to acclimate for at least one week before being used in experiments. Purina Rat Chow and water were available *ad libitum.* The Marshall University Institutional Animal Care and Use Committee approved all animal use in this research (Protocol 531; approved on 1 February 2013). Studies were performed at an AAALAC (Association for the Assessment and Accreditation of Laboratory Animal Care International) accredited facility and all animal care was in accordance with the American Association for Laboratory Animal Science (AALAS) Policy on the Humane Care and Use of Laboratory Animals [65].

### 4.2. Chemicals

All chemicals used were of the highest purity available and were purchased from Sigma Aldrich (St. Louis, MO, USA).

### 4.3. Isolated Renal Cortical Cell (IRCC) Preparation and Treatment

Naive rats were anesthetized with pentobarbital (75 mg/kg, intraperitoneal and isolated renal cortical cells (IRCC) were obtained using the collagenase perfusion method of Jones *et al.* [66]. Cell viability was initially determined by trypan blue (2% *w*/*v*) exclusion and lactate dehydrogenase (LDH) release. IRCC were counted and re-suspended in Krebs-Henseleit buffer, pH 7.4, containing 25 mM Hepes and 2% (*w*/*v*) bovine serum albumin at a concentration of ~4.0 million cells/mL. IRCC (3 mL) were added to a 25 mL polycarbonate Erlenmeyer flask for a 5 min pre-incubation period in a Dubnoff metabolic shaking water bath incubator (GCA Corporation, Precision Scientific Group, Chicago, IL, USA) (37 °C, 60 cycles/min) under a 95% oxygen/5% carbon dioxide atmosphere. IRCC were then exposed to various concentrations of TCA (0, 0.1, 0.25, 0.5, or 1.0 mM) for 60, 90 or 120 min. After the incubation period, flasks were removed and placed on ice. Samples (0.5 mL) were taken for lactate dehydrogenase (LDH) release assays. Briefly, samples were centrifuged (3000× *g*, 3 min) in a Model 59A Microcentrifuge (Fisher Scientific Company, Waltham, MA, USA), the supernatant was decanted and saved, and the pellet was disrupted with 1 mL of 10% Triton X-100 solution to release cellular LDH activity. LDH activity was then determined in each fraction (supernatant and pellet) as previously described using a kinetic assay based on the amount of reduced β-nicotinamide adenine dinucleotide NADH produced from β-nicotinamide adenine dinucleotide (NAD) [30]. LDH released was expressed as % of total (supernatant plus pellet).

In separate experiments, IRCC were pretreated with either an antioxidant or an enzyme system inhibitor before exposure to 0.5 mM TCA for an additional 120 min as described above. The concentrations and pretreatment times for all of the pretreatments are shown in Table 1.

### 4.4. Statistics

Data are presented as mean ± S.E.M. with the number of experiments per assay (*N*) ≥ 4. Data were analyzed by one-way analysis of variance followed by a Student-Newman-Keuls Test. Statistical significance was determined at *p* < 0.05, α = 0.5.

**Table 1:** List of pretreatments and mechanisms/targeted enzyme systems.

Pretreatment	Concentration (mM)	Pretreatment Time (min)	Mechanism of Action
*N*-Acetyl-l-cysteine	2.0	30	Antioxidant
α-Tocopherol	1.0	5	Antioxidant
Glutathione	1.0	30	Antioxidant
Ascorbate	2.0	5	Antioxidant
Methimazole	1.0	30	FMO Inhibitor
*N*-Octylamine	2.0	5	FMO Inhibitor
Indomethacin	1.0	15	Cyclooxygenase Inhibitor
Piperonyl Butoxide	1.0	15	Non-specific CYP Inhibitor
Metyrapone	1.0	5	Non-specific CYP Inhibitor
Mercaptosuccinate	0.1	15	Peroxidase Inhibitor

## 5. Conclusions

The results of this study demonstrate that TCA is toxic to the kidney in a time and concentration dependent manner. Toxicity was evident as early as 90 min with 0.5 mM TCA and with 0.1 mM TCA at 120 min. Since several of the enzyme inhibitor pretreatments attenuated 3,4,5-cytotoxicity, metabolites appear to contribute to the renal toxicity induced by TCA. Bioactivation of TCA to nephrotoxic metabolites via renal CYPs, cyclooxygenase and/or peroxidases, but not FMOs, are potential mechanisms of TCA nephrotoxicity. Free radicals also play a role in TCA nephrotoxicity, as evidenced by the inhibition of TCA cytotoxicity by antioxidants. However, whether the free radicals are intermediates in TCA biotransformation or reactive oxygen species produced during TCA metabolism remains to be determined.
